# Supplementary material for: SyNDI: synchronous network data integration framework
Source: BMC Bioinformatics. 2018 Nov 6;19:403. doi: 10.1186/s12859-018-2426-5 (PMC6219086; doi:10.1186/s12859-018-2426-5)
Supplement: Supplementary file 5 — Files of the example presented in Additional file 4. This additional file is a zip package that contains all files used in Additional file 4. Detailed descriptions of each of these files are presented in a README file included in this zip package. (GZ 30411 kb) [file 12859_2018_2426_MOESM5_ESM.gz › SyNDITools/Meme2Fimo/meme_4.9.0_4.tar.gz!/meme_4.9.0/doc/beadstring.xml]

beadstring - a motif search tool


# beadstring -- not currently supported

**Usage:**

`beadstring [options] <motifs> <database>`

**Description:**

`Beadstring` builds a linear hidden Markov model (HMM) from
the motifs and motif occurences listed in the motif file, and uses
that HMM to search a sequence database for a particular ordered series
of motifs. A description of the algorithm is found in:

Grundy,
Bailey, Elkan and Baker. "Meta-MEME: Motif-based Hidden Markov Models
of Protein Families". *Computer Applications in the
Biosciences.* 13(4):397-406, 1997.

By default, the order and spacing of motifs in the model is determined
from the "Summary of Motifs" section of the MEME input file.
`Beadstring` searches the summary for the sequence that
contains the maximal number of distinct motif occurrences. If there
is a tie, then `beadstring` selects the sequence with the
smallest combined p-value. `Beadstring` then eliminates
all but the most significant occurrence of each motif and uses the
resulting order and spacing of motif occurrences to initialize the
HMM. This procedure can be overridden by selecting the
`--motif`, `--motif-e-thresh`,
`--motif-p-thresh` or `--order` options.

The command line option `--p-score` activates an
alternative scoring mode, called "p-value scoring." This scoring
method is described in

Bailey
and Noble. "Searching for statistically significant regulatory
modules." *Bioinformatics* 19(Suppl 2):ii16-ii25, 2003.

**Input:**

- `<motifs>` is a list of motifs in MEME format.
- `<database>` is a collection of sequences in
  FASTA format.

**Output:**

Beadstring will create a directory, named `beadstring_out` by default.
Any existing output files in the directory will be overwritten.
The directory will contain:

- An XML file named `beadstring.xml` using the
  CisML schema.
- An XML file named `model.xml` using the MEME\_HMM schema.
- An HTML file named `beadstring.html`
- A plain text file named `beadstring.text`

The default output directory can be overridden using the `--o` or `--oc`
options which are described below.

**Options:**

Options related to input and output:

- Default is 0.01.

Options related to selecting motifs for the model:


Options related to building the model:

- Default value is 0.0.
- Default value is 0.0.
- Default value is 0.1.

Options related to scoring:
